# Synbiotic Supplementation Improves Quality of Life and Inmunoneuroendocrine Response in Patients with Fibromyalgia: Influence of Codiagnosis with Chronic Fatigue Syndrome

**DOI:** 10.3390/nu15071591

**Published:** 2023-03-25

**Authors:** María Dolores Hinchado, Carmen Daniela Quero-Calero, Eduardo Otero, Isabel Gálvez, Eduardo Ortega

**Affiliations:** 1 Immunophyisiology Research Group, Instituto Universitario de Investigación Biosanitaria de Extremadura (INUBE), 06080 Badajoz, Spain; 2 Immunophysiology Research Group, Physiology Department, Faculty of Sciences, University of Extremadura, 06071 Badajoz, Spain; 3 Facultad de Deporte, UCAM Universidad Catolica San Antonio de Murcia, 30107 Murcia, Spain; 4 International Chair of Sports Medicine, UCAM Universidad Catolica San Antonio de Murcia, 30107 Murcia, Spain; 5 Immunophysiology Research Group, Nursing Department, Faculty of Medicine and Health Sciences, University of Extremadura, 06071 Badajoz, Spain

**Keywords:** fibromyalgia, chronic fatigue syndrome, synbiotic, inflammation, stress, IL-8, IL-10, cortisol, dehydroepiandrosterone

## Abstract

Fibromyalgia (FM) and chronic fatigue syndrome (CFS) are two medical conditions in which pain, fatigue, immune/inflammatory dysregulation, as well as various mental health disorders predominate in the diagnosis, without evidence of a clear consensus on the treatment of FM and CFS. The main aim of this research was to analyse the possible effects of a synbiotic (Synbiotic, Gasteel Plus^®^ (Heel España S.A.U.), through the study of pro-inflammatory/anti-inflammatory cytokines (IL-8/IL-10) and neuroendocrine biomarkers (cortisol and DHEA), in order to evaluate the interaction between inflammatory and stress responses mediated by the cytokine-HPA (hypothalamic-pituitary-adrenal) axis, as well as mental and physical health using body composition analysis, accelerometry and previously validated questionnaires. The participants were women diagnosed with FM with or without a diagnostic of CFS. Each participant was evaluated at baseline and after the intervention, which lasted one month. Synbiotic intervention decreased levels of perceived stress, anxiety and depression, as well as improved quality of life during daily activities. In addition, the synbiotic generated an activation of HPA axis (physiological cortisol release) that can compensate the increased inflammatory status (elevated IL-8) observed at baseline in FM patients. There were no detrimental changes in body composition or sleep parameters, as well as in the most of the activity/sedentarism-related parameters studied by accelerometry. It is concluded that synbiotic nutritional supplements can improve the dysregulated immunoneuroendocrine interaction involving inflammatory and stress responses in women diagnosed with FM, particularly in those without a previous CFS diagnostic; as well as their perceived of levels stress, anxiety, depression and quality of life.

## 1. Introduction

Fibromyalgia (FM) is a common chronic pain condition, in which patients may also experience a variety of other symptoms, including sleep disturbances, fatigue, stiffness, frequent episodes of pain and mental health problems, as well as possible gastrointestinal disorders [1]. In addition, and according to the American College of Rheumatology [2], such a generalised non-joint pain state occurs for at least three months in duration, predominantly in women over 50 years of age. Chronic fatigue syndrome (CFS) is a condition characterized by persistent and debilitating fatigue lasting at least six months [3]. CFS is the most common comorbidity in patients with FM, ranging from 20–81% [4].

The origin of FM and CFS is unknown, although alterations in the central nervous system, as well as abnormalities in muscle physiology and immune/inflammatory response are suggested as the main causes [5,6,7]. Increasing evidence suggests that the gut microbiota of people suffering from FM and CFS differs from that of healthy individuals, with several studies showing lower values of *Escherichia coli* and *Bifidobacterium* and significantly higher numbers of enterococci compared to healthy controls, potentially leading to various gastrointestinal disorders [8,9]. In addition, several inflammatory and stress biomarkers have been found in our laboratories in previous studies (increased release of pro-inflammatory cytokines including IL-8 together with greater levels of cortisol and noradrenaline in the blood) when comparing FM patients with healthy individuals [6,7,10].

In this context, several food supplements are currently proposed for the improvement of symptoms in FM and CFS, among which we can highlight probiotics [11,12,13,14], as well as synbiotics [15], the latter being very scarce in the literature. Probiotic therapy, according to other authors [16], may change the gut microbiota, enhance mucosal barrier function, reduce pro-inflammatory cytokines and probably have a favourable effect on mood in people who have emotional symptoms and elevated inflammatory immune signals, as well as improvements in cognitive symptoms through neuroimmunoendocrine enhancements [12].

Therefore, the main objective of this research was to analyse the possible effects of a synbiotic in people diagnosed with fibromyalgia through the study of various objective immune/inflammatory and stress biomarkers, as well as perceived mental and physical health parameters, and objective levels of physical activity determined by accelerometry. The existence of a previous diagnosis of chronic fatigue syndrome was also considered to influence some of the parameters evaluated. To the best of our knowledge, this is the first investigation in this context, all this with the aim of helping to a correct prescription of adjuvant treatments that improve the quality of life of these patients, allowing a better differential diagnosis between both syndromes.

## 2. Materials and Methods

### 2.1. The Synbiotic

Within the probiotic strains present in Gasteel Plus^®^ (Heel España S.A.U. laboratories, Madrid, Spain) are *Bifidobacterium lactis* CBP-001010, *Lactobacillus rhamnosus* CNCM I-4036, and *Bifidobacterium longum* ES1, as well as fructooligosaccharides (200 mg) as a prebiotic. Each Gasteel Plus^®^ bar (300 mg) contains 1 × 10^9^ colony forming units (CFU) of freeze-dried powdered bacteria in addition to 1.5 mg zinc, 8.25 g selenium, 0.75 g vitamin D and maltodextrin as an excipient.

### 2.2. Participants

A total of 15 women, diagnosed with FM, with (*n* = 7) or without (*n* = 8) previously diagnosed with CFS. Inclusion criteria included: (i) women between 40–67 years of age, (ii) diagnosed with FM (according to ACR diagnostic criteria) [2], with or without CFS (according to Fukuda and co-workers criteria) [3] by a specialist rheumatologist or internal medicine physician. Participants were excluded if they: (i) consumed any type of probiotic food supplement, (ii) were taking antibiotics, corticosteroids or anti-cytokines therapy during the treatment period, (iii) had a positive medical diagnosis of depression or (iv) were periodically performing scheduled therapeutic physical activity in the two months prior to the accelerometry tests. All participants belonged to a FM association EXISTIMOS ^®^ in Badajoz, Extremadura (Spain). 

Descriptive data of the participants are shown in Table 1. All participants are Caucasian women and have been diagnosed with FM for more than two years (with or without a previous codiagnosis of CFS). No significant differences were found in either age or BMI between the two experimental groups. 

### 2.3. Procedures

This experimental research is part of a pilot study with the aim of identifying the potential benefits of the synbiotic Gasteel Plus^®^ (Heel España S.A.U.) in women diagnosed with FM, with or without a previous diagnostic of CFS. To this end, the subjects were required to maintain a diet similar to the one prior to the treatment period (control diary) and to maintain it during the 30 days of taking the synbiotic. Participants signed an informed consent form prior to the protocol, which was approved in advance by the University of Extremadura committee in accordance with the Council of Europe Directives and the Declaration of Helsinki (registration number 13/2020).

The measurements were performed on two days (baseline and post-test) with a 30-day separation between them in which the participants had to consume the synbiotic, taking one stick a day, preferably in the morning and mixed in water. The order of the tests, the materials used and the members of the research team were the same for the pre-test and post-test so as not to interfere with any of the procedures. Accelerometers and questionnaires were distributed one week before the pre-test and post-test and collected the day of the blood sampling and test determinations.

### 2.4. Biompedance Analysis: Determination of Body Composition Measurements

Body composition was analysed using the BIA TANITA DC-360 digital scale (Tanita, Tokio, Japan), with measurement frequencies between 6.25 kHz/50 kHz. The following data were obtained: weight (kg), % fat mass, body water, muscle mass (kg), bone mass and visceral fat level. BMI was calculated using the formula weight/height^2^ expressed in kg/m^2^. All participants were measured barefoot, lightly clothed and fasting.

### 2.5. Accelerometry: Determination of Objective Levels of Physical Activity, Sedentary Lifestyle and Sleep Quality

The Actigraph wGT3X-BT, a compact and lightweight 3-axis accelerometer (4.6 × 3.3 × 1.5 cm, 19 g) with a response rate of 30–100 Hz, was the accelerometer used in this study. Several objective indicators, including physical activity and intensity, energy expenditure, metabolic equivalent (METs), number of steps taken per week, amount of time spent sitting still, exercise, latency and sleep efficiency are measured by the device. Except at occasions when the accelerometer’s regular operation might be impacted, participants wore an accelerometer fastened to an elastic band on their non-dominant wrist for 7 days in a row (shower or water-related activity). Actilife 6 was used specifically for the analysis of the files the accelerometer produced (ActiGraph, LLC., Pensacola, FL, USA).

### 2.6. Questionnaires: Determination of Perceived Levels of Stress, Anxiety, Fatigue, Pain, Depression, Sleep Quality and Quality of Life

In order to determine the perceived levels of mental health and quality of life of the participants, several scientifically validated questionnaires were used, among them:

- The Spanish version [17] of the Beck Depression Inventory (BDI) was used to determine possible signs of depression in the past week. Higher scores indicate higher levels of depression.
- State-Trait Anxiety Inventory (STAI), to analyse the levels of anxiety presented at a specific time and in general. A Spanish version [18] was used for this purpose. Higher scores indicate higher levels of anxiety.
- The Perceived Stress Scale (PSS), to assess the frequency with which participants experience stressful situations and thoughts in the last month. Higher scores indicate higher levels of stress. Remor was used in its Spanish version [19].
- Brief Pain Inventory (BPI). Used to determine the intensity and interference of pain in daily activities. The greater the perception of pain, the higher the score obtained. A Spanish version [20] was used.
- Brief Fatigue Inventory (BFI). This questionnaire measures the intensity of fatigue in the last 24 h and its interference with daily activities and work. The higher the perception of fatigue, the higher the score obtained [21].
- Healthy Lifestyle and Personal Control Questionnaire (HLPCQ). The Healthy Lifestyle and Personal Control Questionnaire is composed of several sections referring to type of diet, organised physical exercise, as well as social and mental balance [22].
- Pittsburgh Sleep Quality Questionnaire (PSQI). This questionnaire analyses various parameters related to subjective sleep quality: latency, duration, efficiency and disturbances, as well as consumption of sleeping pills. The Spanish version of the questionnaire was used [23].
- FIQ (Fibromyalgia Impact Questionnaire). A Spanish version [24] was used to assess the impact of FM on physical and mental functions (pain, tiredness, fatigue, stiffness, anxiety and depression). Higher scores indicate a worse health condition.
- Gastrointestinal Health Questionnaire. This questionnaire provides insight into gastrointestinal function in adults by identifying the level of severity of gastrointestinal symptoms. The higher the final score, the more severe the symptoms [25].
- COVID-19 questionnaires:
- CAS (Coronavirus Anxiety Scale). The higher the score, the greater the sense of anxiety. Higher scores are related to higher anxiety towards COVID-19 [26].
- FCV-19S (Fear of Coronavirus). The higher the score, the greater the sense of fear of the coronavirus [27].

### 2.7. Blood Sampling: Determination of Inflammatory and Stress Biomarkers

Blood of fasting individuals was extracted at 8 a.m. and placed in collection tubes with EDTA anticoagulant and coagulation agents to separate plasma and serum, respectively. Both the plasma and the serum were centrifuged for 10 min at 1600 and 1800× *g*, respectively. After serum and plasma samples were collected, they were coded and gradually cooled at −20 °C.

For the determination of the pro- and anti-inflammatory cytokines studied (IL-8 and IL-10), the competitive inhibition enzyme-linked immunoassay (ELISA) technique was used using the Human IL-8 and IL-10 Kits (Diaclone Biotech, Besancon, France), respectively. Stress hormones such as cortisol (DetectX^®^ Cortisol enzyme immunoassay kit, Arbor Assays Inc., Ann Arbor, MI, USA) and the hormone dehydroepiandrosterone, DHEA (DEH3344; Demeditec Diagnostics GmbH, Kiel, Germany) were also analysed by ELISA.

### 2.8. Statistics

The statistical analysis was conducted using IBM statistics SPSS v20.0 program. The Shapiro–Wilk test was employed to verify the normality of the data. Student *t*-test paired and unpaired were applied to determine how the intervention affected the outcome. The values were given as mean standard error of mean and the significance threshold was taken into account when *p* < 0.05.

## 3. Results

### 3.1. Effects of the Synbiotic on Body Composition Measurements Determined by Bioelectrical Impedance Analysis (BIA)

The results in Table 2 show that there were no significant changes in weight, body fat mass percentage, bone mass, total body water percentage or muscle mass after consumption of the synbiotic, suggesting that the participants maintained the same diet during the study protocol and that it did not produce any detrimental effect on the body composition of the subjects. Only the visceral fat index decreased statistically significant (*p* < 0.05) in the group of FM patients without a codiagnosis of CFS, but without a physiological relevance.

### 3.2. Effects of the Synbiotic on Physical Activity/Sedentarism Levels and Sleep Quality Determined by Accelerometry

In general, the synbiotic administration did not induce changes in the objective determination of activity/sedentarism and sleep parameters evaluated by accelerometry (Table 3). Only a statistically significant decrease was found in the Total Time in Activity bouts in the total FM patients. This may have been due to the fact that during the month under study, temperatures were quite high and may have reduced the normal physical activity of the subjects.

### 3.3. Effects of the Synbiotic on Perceived Levels of Depression, Stress, Anxiety, Pain, Fatigue, Sleep Quality and Quality of Life Determined by Questionnaires

The results of perceived health measured through previously validated questionnaires are shown in Table 4. Overall, statistically significant improvements were observed in levels of perceived depression, stress, anxiety and fatigue as well as in the fibromyalgia impact on daily activity (*p* < 0.05) in the total group of FM patients. Specifically, depression and stress only statistically improved in FM patients without a codiagnosis of CFS (*p* < 0.05), although anxiety, fatigue and the impact of FM only statistically improved in the FM + CFS group (*p* < 0.05).

Even though without statistical significance, the synbiotic also improved pain, sleep quality and gastrointestinal health of the participants. Furthermore, coronavirus questionnaires show that participants have less fear about contracting the disease in the FM + CFS group.

### 3.4. Effects of the Synbiotic on Inmunoneuroendocrine Biomarkers

#### 3.4.1. Inflammatory Biomarkers (IL-8 and IL-10)

**Figure 1:**
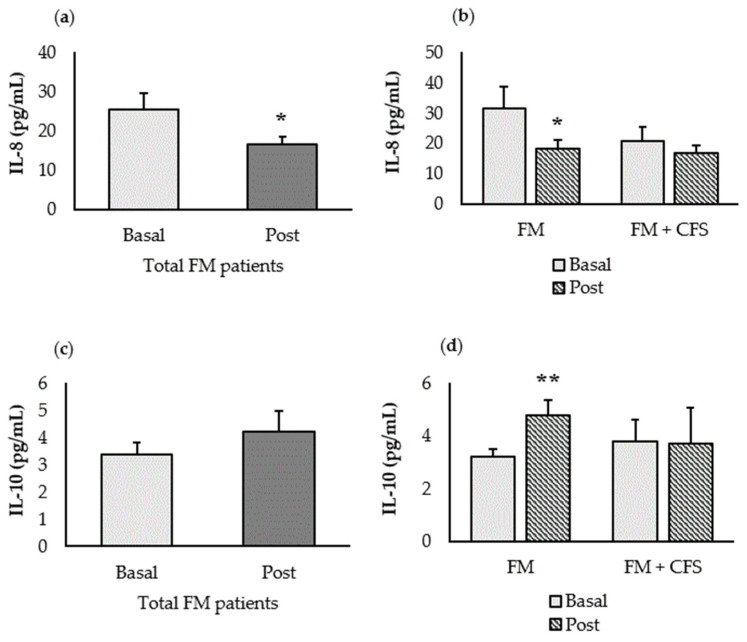
Effect of the synbiotic administration on inflammatory biomarkers. (**a**) IL-8 concentration in the total group of FM patients (*n* = 15), (**b**) IL-8 concentration in the FM patients with (FM + CFS, *n* = 7) or without (FM, *n* = 8) CFS evaluated separately, (**c**) IL-10 concentration in the total group of FM patients (*n* = 15), (**d**) IL-10 concentration in the FM patients with (FM + CFS, *n* = 7) or without (FM, *n* = 8) CFS diagnosed separately. Each column represents the mean ± SEM of the cytokine determination in each patient. * *p* < 0.05 and ** *p* < 0.01 with respect to the baseline.

Figure 1 shows the effect of the synbiotic on inflammatory biomarkers (IL-8 and IL-10) determined by ELISA. After the intervention, FM patients significantly decreased their systemic IL-8 concentration (*p* < 0.05) (Figure 1a). However, we can observe how this significant decrease in IL-8 concentration only occurred in patients without a codiagnosis of CFS (*p* < 0.05) whose baseline levels were above the reference value (>29 pg/mL) of our laboratory in healthy women [6,7] (Figure 1b). Paradoxically, the group with a previous diagnosis of CFS did not present IL-8 levels above the level compatible with healthy individuals.

In addition, the administration of the synbiotic induced an increase (*p* < 0.01) in the anti-inflammatory cytokine IL-10 only in the group of patients without a diagnosis of CFS (Figure 1d). This effect did not occur in the group of FM patients with a previous diagnosis of CFS, which prevented the determination of statistically significant differences in the total group of patients (Figure 1c).

#### 3.4.2. Stress-Related Biomarkers (Cortisol and DHEA)

**Figure 2:**
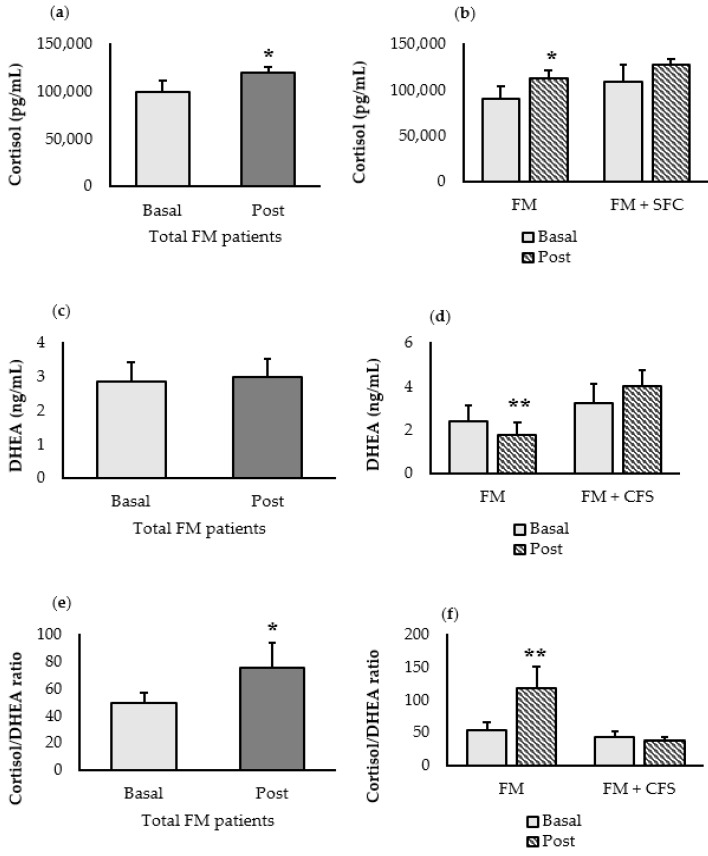
Effect of the synbiotic administration on stress-related biomarkers. (**a**) Cortisol concentration in the total group of FM patients (*n* = 15), (**b**) cortisol concentration in the FM patients with (FM + CFS, *n* = 7) or without (FM, *n* = 8) CFS evaluated separately, (**c**) DHEA concentration in the total group of FM patients (*n* = 15), (**d**) DHEA concentration in the FM patients with (FM + CFS, *n* = 7) or without (FM, *n* = 8) CFS evaluated separately. (**e**) Cortisol/DHEA ratio in the total group of FM patients (*n* = 15), (**f**) Cortisol/DHEA ratio in the FM patients with (FM + CFS, *n* = 7) or without (FM, *n* = 8) CFS evaluated separately. Each column represents the mean ± SEM of the stress-related biomarkers determination in each patient. * *p* < 0.05 and ** *p* < 0.01 with respect to the baseline.

The data obtained for the stress-related hormones, cortisol hormone and dehydroepiandrosterone (DHEA), as well as their ratios are shown in Figure 2. A significant increase (*p* < 0.05) in cortisol is observed after the intervention in the entire group of FM patients (Figure 2a), but this increase (*p* < 0.05), together a decrease (*p* < 0.01) in DHEA concentration, was only found in FM patients without a CFS codiagnosis when evaluated separately (Figure 2b). 

As a consequence, after synbiotic administration, the Cortisol/DHEA ratio increased significantly both in the total group of patients with FM (*p* < 0.05) (Figure 2e) and in the group of patients with FM without previous CFS diagnosis (*p* < 0.01).

## 4. Discussion

FM and CFS are two conditions of diffuse aetiology, often codiagnosed and still poorly differentiated nowadays. Although the preferred symptom in FM is pain and in CFS fatigue, they have many others in common. In fact, studies in our laboratory clearly reveal that, FM patients (with or without a previous diagnostic of CFS) present worse perceived levels of stress, anxiety, fatigue, pain, depression, sleep quality and quality life [28].

Moreover, in recent years, the role of the gut microbiome in host health and disease has been increasingly considered and the so-called gut–brain axis is now clearly accepted. Although patients with FM and CFS frequently have gastrointestinal problems, gut dysbiosis has been described as a consequence rather than a cause of fibromyalgia, with some strains of bacteria overgrowing in the small intestine and, in addition, there seems to be a specific profile of some species in people with FM [29] and with CFS [30]. This alteration of the microbiota is associated with many of the symptoms of FM and CFS, such as chronic widespread musculoskeletal pain [31], fatigue, mood and other symptoms [32]. In addition, after anti-microbial treatments, there seems to be a clinical improvement [33].

Although there are no specific treatments for FM patients, some of the most commonly used strategies to improve the health and quality of life of these patients is through exercise [6,34] and nutrition [13]. Nutrition strategies can acquire particular importance in patients with low adherence to exercise programs due to their difficulties to perform physical activities in long programs. Some supplements studied have included vitamin D [35], iron [36] and magnesium [37]. In addition, as we have stated in previous research, particularly in young sedentary and sports people [38,39], probiotics, prebiotics and synbiotics could be beneficial for health, having positive repercussions on gastrointestinal health, immune and inflammatory response, as well as in other mental health parameters. However, there is little research on the use of probiotics in people diagnosed with FM and/or CFS [12,14,40,41,42,43] and even less after consumption of synbiotics. Thus, very interesting are the results of the present investigation showing that after ingesting a synbiotic for a period of 30 days, FM patients improved their mood, stress and anxiety levels, as well as the impact of the disease on their daily activities subjectively determined by validated questionnaires. These findings could be consistent with other research [44,45] which concludes that by increasing intestinal microbial balance, probiotics improve host health and they may also benefit cognitive and psychological functions via the gut–brain axis [11].

In this context, there is also scientific evidence linking FM and CFS to alterations in the central nervous system [46], which could also be a cause or consequence of the intestinal dysbiosis recently described in patients with FM [32]. Furthermore, FM and CFS are associated with a severely dysregulated immune/inflammatory system, as well as a dysregulation of the HPA (hypothalamic-pituitary-adrenal) axis [6,7,10,34], which may, among others, alter the physical and cognitive health of such individuals [12,28], as previously mentioned. This dysregulation may induce an imbalance in pro- and anti-inflammatory cytokines, with a pivotal role in the pathogenesis of FM [47,48,49]. Probably, an imbalance between pro-inflammatory and anti-inflammatory cytokines leads to chronic peripheral sensitization of the nervous system as a major contributor to pain and the way pain is processed [50,51].

Of these, systemic levels of the pro-inflammatory cytokine IL-8 are the ones frequently seen elevated in rigorously diagnosed FM patients, having even been reported as the best potential biomarker of FM inflammation [6,52,53,54]. Thus, before intervention, the high levels of pro-inflammatory cytokine IL-8 (more than 29 pg/mL as reference value in our laboratory [6,7] have also been determined in FM patients in the present investigation. After the intervention with the synbiotic, systemic levels of IL-8 decreased in FM patients (to values compatible with those of healthy individuals), together with an increase in the systemic levels of the anti-inflammatory cytokine IL-10, all of this particularly evident in the group of FM patients without a previous diagnostic of CFS. 

What is the mechanism used by the synbiotic treatment to regulate the pro-anti-inflammatory cytokine imbalance is the next question that arises. According with the results of the present investigation, some authors [16] proposed that microbiota-induced improve in mucosal barrier function after probiotic administration underly the decrease in pro-inflammatory cytokines that mediate the improvements in emotional and cognitive symptoms via a better inmunoneuroendocrine regulation. In addition, inflammatory and stress responses are bidirectionally regulated. Thus, pro-inflammatory cytokines stimulate the HPA axis, inducing an increase in glucocorticoid levels, which in turn protect the organism from an overproduction of inflammatory cytokines [55]. Disruption of this feedback can aggravate inflammatory conditions, and is found in most underlying autoimmune and inflammatory pathologies, due to a reduced HPA axis response to cytokines or the development of glucocorticoid resistance [55,56], including in FM in which the HPA axis failed to control the increase in pro-inflammatory cytokines [7]. The results presented here support this idea because, after the intervention with the synbiotic, a significant physiological increase in cortisol is observed, together with a decrease in DHEA, particularly in the group without previous diagnosis of CFS, clearly suggesting that the synbiotic generated an activation of the HPA axis (cortisol/DHEA ratio) to compensate for the low-grade inflammation (elevated IL-8) observed in FM patients, also particularly relevant in those without previous diagnosis of CFS. According to some authors [6], the elevated cortisol levels of FM patients is a physiological response to the altered homeostasis caused by their increased inflammatory state. When the stress response is triggered, a negative feedback mechanism is set in motion that protects the body against an “excess” of pro-inflammatory cytokines that can cause tissue damage. This physiological “hormonal” elevation of cortisol following synbiotic consumption may explain the synbiotic induced decrease in IL-8 in the present investigation.

Finally, another question we asked in our research was whether a previous diagnosis of CFS in patients diagnosed with FM could affect the response of the synbiotic. In a previous study conducted in our laboratory CFS codiagnosis does not worsen the subjective perceived psychological and quality of life impairment of FM patients at baseline levels [28]. To the best of our knowledge, this is clearly the first time that the effects of a synbiotic in FM patients (with or without a pre-diagnosis of CFS) have been differentiated. No statistically significant changes were observed in most body composition parameters and physical activity levels during the month-long protocol, as measured objectively by accelerometry. These results suggest that the consumption of the synbiotic did not negatively affect the body composition of the participants during this short period of time, coinciding with another study in which a synbiotic containing probiotics and inulin as a prebiotic was administered [15]. Nevertheless, while the improvement in perceived parameters after taking the synbiotic, such as stress and depression, was more evident in the FM group without a pre-diagnosis of CFS, in perceived fatigue, trait anxiety and fear of COVID-19, the improvement was more evident in the group of FM patients with a pre-diagnosis of CFS. However, as stated before, objective results related to a better regulation of the cytokine-HPA-axis induced by the synbiotic was only found in the group of FM patients without a previous diagnostic of CFS (also with basal elevated levels of IL-8); probably suggesting that women with CFS are, sometimes, over-diagnosed with FM via subjective and perceived evaluations.

A limitation of the present investigation has been the lack of evaluation of the basal level of dysbiosis of FM patients and if the 30 days intervention with the synbiotic is enough to really change the microbiota. Further studies could consider measuring some of the strains characteristic of these two syndromes, before and after treatment with the synbiotic, in order to verify whether a modification of the existing dysbiosis could really be the mechanism responsible for the improved immunoneuroendocrine regulation seen in these patients after consumption of the synbiotic. Although the fact that all the patients who met the inclusion criteria were accepted in the study reinforces the results obtained, future studies with larger numbers of participants, a longer intervention seems to be necessary, particularly found more clearly statistical differences when evaluating FM with or without codiagnosis of CFS separately.

## 5. Conclusions

In conclusion, the synbiotic seems to have a beneficial effect on the inmunoneuroendocrine imbalance presented by women with FM, provoking a clear response of activation of the HPA axis and subsequently a decrease in the inflammatory profile, an effect that only occurs in patients without a previous diagnosis of CFS. In addition, it produces significant improvements in perceived levels of stress, anxiety and depression, as well as improvements in quality of life during daily activities.

## Figures and Tables

**Table 1:** Descriptive data of the participants.

Variable	Total FM Patients (*n* = 15)	FM (*n* = 8)	FM + CFS (*n* = 7)
Gender (%)	Women (100%)	Women (100%)	Women (100%)
Ethnic group (%)	Caucasian (100%)	Caucasian (100%)	Caucasian (100%)
Duration of FM/CFS diagnosed (years)	>2	>2	>2
Age (years)	59.38 ± 2.35	63.00 ± 3.35	55.75 ± 3.18
BMI (kg/m^2^)	29.19 ± 1.50	29.78 ± 1.35	28.60 ± 2.82

The data are represented as mean ± SEM. BMI: Body Mass Index; CFS: Chronic Fatigue Syndrome; FM: Fibromyalgia.

**Table 2:** Results of body composition measurements determined by Bioelectrical Impedance Analysis (BIA).

	Total FM Patients (*n* = 15)		FM (*n* = 8)		FM + CFS (*n* = 7)	
	Basal	Post	Basal	Post	Basal	Post
Weight (kg)	76.93 ± 3.76	76.72 ± 3.76	75.33 ± 2.47	74.56 ± 2.47	78.76 ± 7.91	79.19 ± 7.62
Body fat mass (%)	40.15 ± 1.55	40.43 ± 1.54	41.25 ± 1.55	41.50 ± 1.53	39.05 ± 2.80	39.36 ± 2.79
Bone mass (kg)	2.30 ± 0.05	2.30 ± 0.06	2.33 ± 0.03	2.30 ± 0.05	2.28 ± 0.11	2.30 ± 0.11
Body water (%)	41.25 ± 1.03	41.34 ± 1.05	40.29 ± 1.42	40.29 ± 1.44	42.21 ± 1.59	42.40 ± 1.62
Muscle mass (kg)	42.78 ± 1.09	42.83 ± 1.07	42.99 ± 0.87	42.61 ± 1.08	42.56 ± 2.10	43.05 ± 1.98
Visceral fat index	9.53 ± 0.87	9.56 ± 0.75	10.31 ± 0.80	10.13 ± 0.81 *	8.75 ± 1.45	9.00 ± 1.33

* *p* < 0.05 indicate statistically significant difference with respect to the BASAL values. The data are represented as mean ± SEM. CFS: Chronic fatigue Syndrome; FM: Fibromyalgia.

**Table 3:** Objective results of physical activity, sedentary levels and sleep quality determined by Accelerometry.

	Total FM Patients (*n* = 15)		FM (*n* = 8)		FM + CFS (*n* = 7)	
	Basal	Post	Basal	Post	Basal	Post
METs (mL O_2_/kg·min)	1.43 ± 0.04	1.41 ± 0.04	1.47 ± 0.06	1.44 ± 0.05	1.37 ± 0.04	1.36 ± 0.05
Activity bouts (<1 min)	58.04 ± 7.59	49.10 ± 7.19	58.71 ± 11.08	54.70 ± 10.62	58.25 ± 9.81	39.25 ± 6.22
Total Time in Activity bouts (min)	974 ± 166.38	759.36 ± 123.17 *	1004.71 ± 251.04	847.86 ± 179.17	921.75 ± 178.188	604.5 ± 118.98
Average Time per Activity bout (min)	15.55 ± 0.98	14.87 ± 0.62	16.05 ± 1.53	15.00 ± 0.74	14.84 ± 1.12	14.68 ± 1.19
Sedentary bouts (<1 min)	125.01 ± 7.95	120.67 ± 8.17	124.28 ± 12.58	119.14 ± 11.02	126.01 ± 9.19	122.8 ± 13.57
Total Time in Sedentary bouts (min)	2885.67 ± 235.55	2599.91 ± 155.15	2860.85 ± 335.14	2561.57 ± 219.28	2920.41 ± 360.31	2653.61 ± 238.06
Average Time per Sedentary bout (min)	23.22 ± 1.43	21.84 ± 0.72	22.95 ± 1.02	21.80 ± 0.92	23.61 ± 3.36	21.90 ± 1.23
Sleep latency (min)	0.70 ± 0.14	0.75 ± 0.14	1.14 ± 0.56	1.14 ± 0.86	0.91 ± 0.25	0.72 ± 0.22
Sleep efficiency (%)	87.71 ± 1.37	87.09 ± 1.37	88.72 ± 1.73	87.79 ± 2.03	86.29 ± 2.30	86.1 ± 1.49
WASO (min)	49.18 ± 5.25	53.37 ± 6.21	45.36 ± 6.15	49.95 ± 9.44	54.54 ± 9.50	58.16 ±7.61

* *p* < 0.05 indicate statistically significant difference with respect to the BASAL values. The data are represented as mean ± SEM. CFS: Chronic fatigue Syndrome; FM: Fibromyalgia; METs: Metabolic Equivalent of Task; WASO: Wakefulness After Sleep Onset.

**Table 4:** Results of perceived levels of stress, anxiety, fatigue, pain, depression, sleep quality and quality of life.

	Total FM Patients (*n* = 15)		FM (*n* = 8)		FM + CFS (*n* = 7)	
	Basal	Post	Basal	Post	Basal	Post
Healthy Life and Personal Control Score	63.27 ± 3.38	65.40 ± 3.21	66.13 ± 3.42	69.00 ± 3.99	60.00 ± 6.19	61.29 ± 4.99
Beck’s Depression Score	18.67 ± 2.77	15.67 ± 2.82 *	18.13 ± 3.30	14.75 ± 3.24 *	19.29 ± 4.86	16.71 ± 5.03
Perceived Stress Score	31.27 ± 3.02	26.87 ± 2.89 *	31.75 ± 3.40	25.75 ± 3.26 *	30.71 ± 5.48	28.14 ± 5.19
Trait-Anxiety Score	33.93 ± 3.74	31.40 ± 3.51 *	34.38 ± 4.98	31.88 ± 4.78	33.43 ± 6.06	30.86 ± 5.59 *
State-Anxiety Score	33.80 ± 4.33	31.53 ± 4.10	34.25 ± 5.23	31.25 ± 5.25	33.29 ± 7.55	31.86 ± 6.89
Brief Pain Inventory Score	6.24 ± 0.46	5.97 ± 0.46	6.57 ± 0.41	6.22 ± 0.37	5.88 ± 0.87	5.68 ± 0.91
Brief Fatigue Inventory Score	7.28 ± 0.40	6.52 ± 0.57 *	6.95 ± 0.51	6.66 ± 0.41	7.65 ± 0.65	6.35 ± 1.18 *
Pittsburgh Sleep Quality Score	12.60 ± 1.02	11.73 ± 0.81	11.13 ± 0.81	10.25 ± 0.41	14.29 ± 1.86	13.43 ± 1.46
Coronavirus Anxiety Score	2.07 ± 0.85	1.47 ± 0.60	2.88 ± 1.44	1.88 ± 0.97	1.14 ± 0.77	1.00 ± 0.69
Fear of COVID-19 Score	13.85 ± 2.06	13.73 ± 1.73	14.33 ± 4.01	15.50 ± 2.77	13.43 ± 2.03	11.71 ± 1.84 *
Fibromyalgia Impact Questionnaire Score	55.37 ± 3.09	50.51 ± 3.31 *	54.55 ± 3.98	51.35 ± 4.01	56.30 ± 5.13	49.56 ± 5.74 *
Gastrointestinal Health Score	9.73 ± 1.39	9.53 ± 1.41	11.13 ± 1.88	10.88 ± 2.29	8.14 ± 2.04	8.00 ± 1.48

* *p* < 0.05 indicate statistically significant difference with respect to the BASAL values. The data are represented as mean ± SEM. CFS: Chronic fatigue syndrome; FM: Fibromyalgia.

## Data Availability

The raw data supporting the conclusions of the manuscript will be made available by the authors, without undue reservation, to any qualified researcher.
